# Structure and Ligand Based Drug Design Strategies in the Development of Novel 5-LOX Inhibitors 

**DOI:** 10.2174/092986712801661112

**Published:** 2012-08

**Authors:** Polamarasetty Aparoy, Kakularam Kumar Reddy, Pallu Reddanna

**Affiliations:** 1 School of Life Sciences, University of Hyderabad, Hyderabad – 500046, India; 2 National Institute of Animal Biotechnology, Hyderabad, India

**Keywords:** Arachidonic acid, 5-LOX, asthma, drug design, pharmacophore, QSAR, scaffold hopping, pseudoreceptor.

## Abstract

Lipoxygenases (LOXs) are non-heme iron containing dioxygenases involved in the oxygenation of polyunsaturated fatty acids (PUFAs) such as arachidonic acid (AA). Depending on the position of insertion of oxygen, LOXs are classified into 5-, 8-, 9-, 12- and 15-LOX. Among these, 5-LOX is the most predominant isoform associated with the formation of 5-hydroperoxyeicosatetraenoic acid (5-HpETE), the precursor of non-peptido (LTB_4_) and peptido (LTC_4_, LTD_4_, and LTE_4_) leukotrienes. LTs are involved in inflammatory and allergic diseases like asthma, ulcerative colitis, rhinitis and also in cancer. Consequently 5-LOX has become target for the development of therapeutic molecules for treatment of various inflammatory disorders. Zileuton is one such inhibitor of 5-LOX approved for the treatment of asthma.

In the recent times, computer aided drug design (CADD) strategies have been applied successfully in drug development processes. A comprehensive review on structure based drug design strategies in the development of novel 5-LOX inhibitors is presented in this article. Since the crystal structure of 5-LOX has been recently solved, efforts to develop 5-LOX inhibitors have mostly relied on ligand based rational approaches. The present review provides a comprehensive survey on these strategies in the development of 5-LOX inhibitors.

## INTRODUCTION TO DRUG DESIGN

### 5-LOX and its Importance

Arachidonic acid (AA) is normally found esterified to cell membrane glycerophospholipids. Activation of phospholipase A_2_ (PLA_2_) results in the release of AA from membrane phospholipids and makes it available for oxidative metabolism by several enzyme systems. AA can be metabolized by three pathways: cyclooxygenase (COX), lipoxygenase (LOX) and epoxygenase (EPOX) as depicted in Fig. (**1**).

COXs (prostaglandin-endoperoxide synthase, EC 1.14.99.1) catalyze the production of prostaglandins (PGs), prostacyclins and thromboxanes (TXs). The COX activity introduces two molecules of oxygen into AA to form the cyclic hydroperoxy endoperoxide (PGG_2_), which is subsequently reduced by the peroxidase to the hydroxy endoperoxide, PGH_2 _[1]. There are three isoforms COX-1, COX-2, and COX-3 [2]. COX-1, constitutively expressed in most tissues and involved in the synthesis of prostaglandins (PGs) at low levels, is presumed to function primarily in the maintenance of physiological functions [3-5]. COX-2, the inducible isoform of COX, is induced by several mitogenic and proinflammatory stimuli and plays a direct role in tumor cell growth and various other diseases. COX-3 is recently identified isozyme and is a splice variant of COX-1.

LOXs (linoleate: oxygen oxido reductase, EC 1.13.11.12) are a group of closely related non-heme iron containing dioxygenases. These enzymes catalyze the addition of molecular oxygen into Poly Unsaturated Fatty Acids (PUFAs) containing cis, cis 1-4 pentadiene structures to give their hydroperoxy derivatives [6]. All LOXs have a two domain structure, the small N-terminal β-barrel domain and larger catalytic domain containing non-heme iron atom. They contain a ‘‘non-heme’’ iron per molecule in the active site as high-spin Fe(II) in the native state, and high-spin Fe(III) in the activated state [7-8]. Iron is ligated in an octahedral arrangement by three conserved histidines, one His/Asn/Ser, and a conserved isoleucine at the C-terminus of the protein [9]. LOX proteins have a single polypeptide chain with a molecular mass of 75–80 kDa in animals and 94–104 kDa in plants and the highest sequence identity between these LOXs is in the portion of the catalytic domain near the iron atom [10]. 

LOXs are classified on the basis of site of arachidonate oxygenation into 5-, 8-, 9-, 11-, 12- and 15-LOX. Though most of the lipoxygenases insert molecular oxygen stereospecifically at ‘S’, recently ‘R’ lipoxygenases also have been reported [11-15]. The prominent animal LOXs are 5-LOX, 8-LOX, 12-LOX and 15-LOX, while the plant LOXs are mostly 5-LOX and 15-LOX. Among these, 5-LOX is the most predominant isoform associated with the formation of 5-hydroperoxyeicosatetraenoic acid (5-HpETE) and other bioactive lipid mediators [16]. Cellular activation by immune complexes and other inflammatory stimuli result in an increase in intracellular calcium and the translocation of Cytosolic Phospholipase A_2_ (cPLA_2_) and 5-LOX from the cytosol to the nuclear membrane and association with 5-lipoxygenase activating protein (FLAP), an 18-kDa integral membrane protein essential for Leukotriene (LT) biosynthesis in intact cells. FLAP selectively transfers AA to 5-LOX and enhances the sequential oxygenation of AA to 5-HpETE and dehydration to LTA_4_ [17-21]. LTA_4_ can be further metabolized to LTB_4_ by LTA_4_ hydrolase or to LTC_4_ by conjugation of glutathione at the sixth carbon by the action of LTC_4_ synthase [20]. Additional studies established that LTC_4_ and its extracellular metabolites LTD_4_ and LTE_4_ are the constituents of slow-reacting substance of anaphylaxis, but they are now more properly termed as cysteinyl leukotrienes. The cysteinyl leukotrienes have been recognized to mimic many of the clinical manifestations of asthma. LTE_4_ is further metabolized to inactive LTF_4_ by the action of c-glutamyl transpeptidase. Studies have also shown that LTF_4_ was formed directly from LTC_4_ by the action of carboxypeptidase [22]. LTB_4_ is a potent chemotactic and chemokinetic agent for a variety of leukocytes, the cysteinyl leukotrienes C_4_, D_4_ and E_4_ cause vascular permeability and smooth muscle contraction [23].

LTs are involved in a variety of inflammatory and allergic diseases such as asthma, ulcerative colitis and rhinitis [14]. 5-LOX pathway is also associated with gastroesophageal reflux disease (GERD) and Crohn's disease [24]. The potential role of leukotrienes in atherosclerosis, another chronic inflammatory disease has been recently discussed [25]. 5-LOX plays an important role in distinct types of cancers like colon, esophagus, prostate, lung, etc. [26-30]. Recently it has also been shown that 5-LOX (ALOX5) is critical regulator for leukemia cancer stem cells (LSCS) in chronic myeloid leukemia (CML) [31]. 

It plays role in tumorigenesis, mainly in stimulating cell proliferation; genotoxicity; inhibition of apoptosis and in increased metastasis and angiogenesis [32]. There are numerous reports on over expression of 5-LOX in cancer cells and the protective role of its inhibitors. Hong *et al. *[33] have shown that 5-LOX and 5-LOX activating protein (FLAP) were universally expressed in epithelial cancer cell lines. Further in another study, Hennig *et al. *[34], demonstrated over expression of 5-LOX in human pancreatic cancer cells. Gupta *et al. *have reported overexpression of 5-LOX protein in malignant tissue in patients with prostate carcinoma. They reported 2.2 fold greater levels of 5-HETE in malignant tumor tissue compared with benign tissue suggesting the clinical importance of 5-LOX inhibitors in patients with prostate carcinoma [35]. MK591, a specific inhibitor of 5-LOX activity was shown to induce apoptosis in prostate cancer cells *via* down-regulation of PKCε, a pro-survival serine/threonine kinase [36-37]. 

Studies have shown that both 5-LOX and 12-LOX mRNA and protein are expressed in human pancreatic cancer cell lines but not in normal human pancreatic ductal cells [38]. In another study MK886, a selective inhibitor of 5-LOX which does not inhibit COX-2 and 12-LOX, was shown to inhibit celecoxib-induced increase in 5-LOX gene expression and Erk1/2 activation in pancreatic cell line SW1990 cells. It was observed that combined use of the COX-2 inhibitor, celecoxib and 5-LOX inhibitor, MK886 is a highly effective way to suppress the growth of human pancreatic cancer cell line SW1990 [39].

Hoque *et al. *have demonstrated that 5-LOX protein expression is increased in esophageal cancer and that 5-LOX inhibitors caused a dose- and time-dependent induction of apoptosis [40]. Zou *et al.* have reported significantly higher levels of 5-LOX mRNA and protein in gastric cancer than in non-tumor tissues and have shown that 5-LOX selective inhibitor AA861 induces apoptosis in human gastric cancer AGS cell line [41]. Recently, Melstrom *et al.* have shown that 5-LOX is upregulated in adenomatous colon polyps and cancer when compared with normal colonic mucosa and they have revealed that the inhibition of 5-LOX in an *in vivo *colon cancer xenograft inhibited tumor growth [42], showing the importance of 5-LOX inhibitors in colon cancer patients. In our recent reports, we demonstrated anti proliferative effects of 5-LOX inhibitors in various cancer cell lines [43-44].

Recent studies have shown that the adverse reactions in patients taking COX inhibitors are due to the shunting of LT synthesis through 5-LOX pathway [45-48]. Studies have also shown that treatment with 5-LOX inhibitor MK886 increases prostaglandin E_2_ production in colon cancer cells [49], suggesting that blocking one metabolic pathway can shunt the AA metabolism toward the other pathway. Earlier reports suggested that the macrophages from 5-LOX knockout mice compensate for the inability to synthesize leukotrienes by upregulating prostaglandins and thromboxane biosynthesis [50]. In another study, 5-LOX knockout mice exhibited significantly greater levels of PGE_2_ than WT mice in lung lavage fluid of bleomycin-treated mice but this was observed only at Day 7 and later time points [51]. Since blocking one of the AA metabolizing pathways (e.g., COX-2) may activate other pathways (e.g., 5-LOX) or vice versa additive effects of these inhibitors is also of great interest. There is evidence that combined use of COX-2 and 5-LOX inhibitors produce additive antitumor effects in colon cancer [49-52]. Li *et al.* [53] have shown over expression of 5-LOX and COX-2 in hamster and human oral cancer and demonstrated synergistic effects of zileuton and celecoxib, inhibitors of 5-LOX and COX-2 pathways respectively. Previously additive effect of these inhibitors was shown in human esophageal adenocarcinoma [54]. Licofelone, a potent COX-2/5-LOX inhibitor was shown to induce apoptosis in both androgen-dependent and androgen-independent prostate [55] and colon [56] cancer cells. Recent reports have shown that Licofelone-nitric oxide donors exhibit high antiproliferative potency in breast cancer as well as in colon cancer cells [57]. In our recent study, Chebulagic acid, a COX-LOX dual inhibitor was isolated from the fruits of Terminalia chebula Retz. It showed antiproliferative activity against HCT-15, COLO-205, MDA-MB-231, DU-145 and K562 cell lines [58-59].

There is increasing evidence of role of 5-LOX products in various diseases, the inhibitors of 5-LOX therefore have great therapeutic potential. 5-LOX inhibitors belong to four distinct classes, namely, (1) redox inhibitors (2) iron chelators (3) competitive reversible inhibitors, and (4) inhibitors of FLAP. In his review, Young has discussed the status of 5-LOX inhibitors and stated that despite intensive efforts towards 5-LOX inhibitor development, only the iron chelating inhibitors of 5-LOX have been successful so far [60]. Of the many 5-LOX inhibitors identified only zileuton (1-(1-benzothiophen-2-ylethyl)-1-hydroxy-urea) could enter the market in 1996 as 5-LOX inhibitor for the treatment of asthma [61]. However, it is not widely prescribed for asthma because of a high dose regimen (600 mg q.i.d. or 1200 mg b.i.d.), low but significant hepatotoxicity [62-64]. Most of the other potent inhibitors of 5-LOX in cellular level often markedly lost efficacy or showed toxic side effects in whole blood, animal studies or human clinical trials. Selective inhibitors of 5-LOX inhibitor, AA-861 and ZD2138 have been discontinued in Phase II clinical trials [64]. In their respective review articles, Young [60] and Steele [64] have discussed the fate of various 5-LOX inhibitors in clinical trials. 

Some of the 5-LOX inhibitors developed are in clinical trials for various diseases. PEP03, developed by PharmaEngine is a new chemical entity developed as a highly selective, potent, and orally active 5-LOX inhibitor and studies to assess the efficacy and safety of treatment with PEP03 in patients with chronic obstructive pulmonary disease (COPD) is going on. A new 5-LOX inhibitor, derived from a herb, has undergone a phase II trial in osteoarthritis with promising results [65]. VIA-2291, a selective and reversible inhibitor of 5-LOX is in phase III for its effects on atherosclerotic plaque, an underlying cause of heart attack, stroke and other vascular diseases [66]. Apart from these, the efficacy of addition of adjuvant from Boswellia serrata, a selective anti-inflammatory herbal medicine and 5-LOX Inhibitor identified by The Cleveland Clinic and National Cancer Institute (NCI) is in Phase II of trail as an adjuvant therapy in newly diagnosed and recurrent high-grade gliomas. 

The association of products of 5-LOX in numerous diseases makes it a very promising therapeutic target. Consequently an emerging strategy consists of creating molecules with specific 5-LOX inhibition activity and fewer or no side effects.

### Types of Drug Design

Drug discovery and development is very expensive and time consuming process. Traditional approaches to drug discovery rely on a step-wise synthesis and screening of large number of compounds to identify a potential candidate. Over the past ten to twenty years, there is an increased effort to apply computational power to the combined chemical and biological space in order to streamline drug discovery, design, development and optimization [67]. Computational methods are expected to play an imperative role in understanding the specific molecular recognition events of the target macromolecule with candidate hits leading to the design of improved leads for the target [68]. Computer Aided Drug Design (*in silico*) approaches have been widely employed in Lead Identification and Lead Optimization stages of drug development against various targets over the years. In comparison to traditional drug discovery methods rational drug design methods bring down the time and cost involved in drug development process. It can be used to identify/design new inhibitors *de novo *or for optimization of absorption, distribution, metabolism, excretion and toxicity profile of identified molecules from various sources. Advances in computational techniques and hardware have facilitated the application of *in silico *methods in the discovery process. Drug Design can be categorized as two types: Structure based drug design (SBDD) and Ligand based drug design (LBDD).

### Structure Based Drug Design

SBDD is the approach where the structural information of the drug target is exploited for the development of its inhibitor. Receptor structure(s) is a prerequisite for this method. Most commonly the structure of the receptor is determined by experimental techniques such as X-ray crystallography or NMR. If the structure of the protein drug target is not available, protein structure can be predicted by computational methods like threading and homology modeling. Threading (also called as fold) is a modeling approach used to model proteins that do not have homologous proteins with known structure. During threading, a given amino acid sequence is searched for compatibility with the structures in a database of known folds. The structure of the query protein is built from these folds. Homology modeling (also called as comparative) is an approach that relies on a clear relationship or homology between the sequence of the target protein and at least one known structure [69]. The process of homology modeling of proteins consists of the following steps: Identification of homologous protein with known 3D structure(s) that can serve as template; sequence alignment of target and template proteins; generation of model for the target based on the 3D structure of the template and the alignment; model refinement and validation [70-71]. Over the years, homology modeling has become the main alternative to get a 3D representation of the target in the absence of crystal structures.

#### De Novo Drug Design

*De novo* is a Latin expression meaning "from the beginning". Active site of drug targets when characterized from a structural point of view will shed light on its binding features. This information of active site composition and the orientation of various amino acids at the binding site can be used to design ligands specific to that particular target. Computational tools that can analyze protein active site and suggest potential compounds are extensively used for *de novo* design methods. Many promising approaches with the goal of ligand design have been reported. In his book chapter, Murcko [72] provided a detailed analysis of computer aided ligand design methods and distinguished them as six major classes as shown in Fig. (**2**):

- Fragment location methods: To determine desirable locations of atoms or small fragments within the active site.
- Site point connection methods: To determine locations (“site points”) and then place fragments within the active site so that those locations are occupied by suitable atoms.
- Fragment connection methods: Fragments are positioned and “linkers” or “scaffolds” are used to connect those fragments and hold them in a desirable orientation.
- Sequential buildup methods: Construct a ligand atom by atom, or fragment by fragment.
- Whole molecule methods: Compounds are placed into active site in various conformations, assessing shape and/or electrostatic complementarity.
- Random connection methods: A special class of techniques combining some of the features of fragment connection and sequential buildup methods, along with bond disconnection strategies and ways to introduce randomness.

Over the years various *de novo* methods especially whole molecule methods like docking have become integrated within disciplines that include chemistry, pharmacology, molecular biology and computer modeling. Electrostatic and solvation terms critical for evaluating correct binding energies, are difficult and slow to calculate. Advances in algorithm sophistication are providing better and better approximations for these parameters [73]. Finally, it is clear from the recent literature that the drug design process has become an essential part of drug discovery projects.

#### Structure Based Virtual Screening

Structure based virtual screening is one of the commonly used approaches in lead identification step and is seen as a complementary approach to experimental high throughput screening (HTS) to improve the speed and efficiency of the drug discovery and development process [74]. This involves explicit molecular docking (process to predict binding mode) of each ligand to the binding site of the target and scoring (process to measure binding affinity). The compounds in the databases screened are ranked with a view to selecting and experimentally testing a small subset for biological activity considered to be appropriate for a given receptor [69,75-76]. Many successful applications have been reported in the field of molecular docking based virtual screening [77-83]. Although the energy calculations involved are crude, the compounds in the library are readily available, making experimental testing easy and false positives tolerable [76].

### Ligand Based Drug Design

Ligand based drug design is an approach used in the absence of the receptor 3D information and it relies on knowledge of molecules that bind to the biological target of interest. 3D quantitative structure activity relationships (3D QSAR) and pharmacophore modeling are the most important and widely used tools in ligand based drug design. They can provide predictive models suitable for lead identification and optimization [84]. Further information on these methods and their application to 5-LOX inhibitor design and development are presented elsewhere in the review.

## APPLICATION OF SBDD IN 5-LOX INHIBITOR DEVELOPMENT 

Structure based methods have not played a major role when compared to other drug targets in the discovery of 5-LOX inhibitors as the crystal structure of 5-LOX has been solved very recently [85]. Various research groups have used homology modeling technique for the generation of reasonable 3D model of 5-LOX which were in turn used to understand the binding site features and SAR of known inhibitors. Du *et al.*, (2006) developed 3D model of 5-LOX using rabbit 15-LOX as the template and performed molecular docking simulation analyses to predict binding free energies for the inhibitors [86]. AutoDock 3.0.3 was used for docking and to calculate the binding free energy between ligand and receptor and estimate Ki value through it’s empirically calibrated score function. K_D _values were measured by SPR and values obtained correlated very well with the biological activities with a high correlation (R^2^) of 0.814. The binding energies obtained from docking studies were also in agreement with experimental data hence supporting the correctness of the constructed 3D model. The molecular docking methodology applied provided a reasonable and reliable 5-LOX/inhibitor binding model which has potential for application in the structure-based discovery of novel 5-LOX inhibitors. Docking studies of the inhibitors have shown that hydrogen bonds and hydrophobic interactions play a key role in inhibitor binding at the enzyme active site. They attributed the lack of a hydrophobic body to the low affinity of phenidone towards 5-LOX.

In their studies, Charlier *et al.*, (2006) generated the 3D structure of human 5-LOX based on the crystal structure of rabbit 15-LOX and studied the binding modes of a set of competitive inhibitors of 5-LOX [87]. They pointed out that the modeled active site of human 5-LOX is more spacious than that of rabbit 15-LOX and consists of a deep bent-shaped cleft containing the non-heme iron cofactor. It was identified that the main binding cleft extends from Phe177 and Tyr181 in the upper part to Trp599 and Leu420 at the bottom. Arg411, Tyr181, Leu414, Asn425, Arg411, and Phe421 were shown to be important, capable of strong interactions with the ligand at active site.

There are limitations in obtaining sufficient quantity of the purified 5-LOX in stable form from mammalian sources. As a result potato tuber 5-LOX, with similar catalytic activity, is frequently employed as the abundant source [88] and in screening specific inhibitors. Hence we developed 3-D model of potato 5-LOX based on the known structure of soybean lipoxygenase-3 complexed with 4-nitrocatechol (PDB ID:1NO3 [89]) [90]. The conserved structural pattern of all LOXs, mainly at the catalytic site, provided an advantage for building up an accurate homology model. In our study, we modeled 5-LOX, performed flexible docking with already reported inhibitors and further calculated relative binding energies of docked inhibitors using energy minimization calculations. Polar residues like Gln521, Glu787, His530, His525, His271, His783, Thr784, Arg782, Asp276, Arg559, Asp560, Asn563, Asn565, Asn720, Ser567, Ser863 and Thr279 were found distributed along the 5-LOX active site channel. Charged amino acids Asp276, Asp560, His525 and His530 are present in the active site channel and may play vital role in ligand binding [91]. Docking studies have shown that the inhibitors bind firmly to the open cavity that is in the sixth coordination of the iron atom and may thus prevent the access of the substrate to the catalytic site of 5-LOX. The relative binding energy results correlated well with the experimental data and supported the model. 

In another study, we designed mono- and di-*O*-prenylated chalcone derivatives using the homology model of 5-LOX [45]. The molecules were docked using GOLD (Genetic Optimization of Ligand Docking), a docking program based on genetic algorithm [92]. After docking, the individual binding poses of each ligand were observed and their interactions with the protein were studied. The best and the most energetically favorable conformation of each ligand were selected and complexed with 5-LOX model. A four-stage protocol was set up for energy minimizations of the protein-inhibitor complex using AMBER [93]. After four stage protocol energy minimizations, relative binding energies were calculated. 

The LUDI scoring method [94] of interactions between a protein and its ligand was also used in this study to quantify the binding affinity of the compounds to 5-LOX. LUDI score is calculated using Eq.** 1**.

(1) *LUDI *Score = -73.33 mol/kcal ΔG

ΔG = ΔG_o_ + ΔG*_hb_f*(ΔR)*f*(Δa) + ΔG*_ion_f*(ΔR)*f*(Δa) + ΔG_*lipo*_A*_lipo_* + ΔG_*rot*_NR ΔG;

ΔG_o_ represents the contribution to the binding energy that does not directly depend on any specific interactions with the receptor, 

ΔG_*hb*_ and ΔG_*ion*_ represent the contribution from an ideal hydrogen bond and unperturbed ionic interactions respectively, 

ΔG_*lipo*_ represents the contribution from lipophilic interactions which is proportional to the lipophilic surface A*_lipo_*, 

ΔG_*rot*_NR represents the contribution due to freezing of internal degrees of freedom in the fragment, 

NR is the number of acylic bonds, ΔR is the deviation of the hydrogen bond length from the ideal value of 1.9 Å, 

Δa is the deviation of the hydrogen bond angle from the ideal value of 180°.

In general, a higher LUDI score (0-1100 in range) represents higher affinity and stronger binding of a ligand to the receptor. The di**-***O***-**prenylated chalcones were predicted to be more effective than their mono**-***O***-**prenylated analogues. A comparison of SAR data showed that the LUDI score increased with increase in the number of hydrogen bonds increase. In the case of the most highly scored compound, Mol-1, most of the functional groups interacted favorably with the 5-LOX active site. Strong hydrogen bond interactions were observed between 3-methoxy of, Mol-1 and Thr 784 (O^…..^HO, 3.7 Å), 4-methoxy and His 271 (O^…..^HN, 2.4 Å) and 5-methoxy and Asp 560 (O^…..^HO, 2.3 Å) respectively. The prenyl group of the 2´*O*-prenyl group formed strong hydrophobic interactions with Leu 255 and Lys 283 along with strong hydrogen bond interactions between oxygen atom of 2´*O*-prenyl and Ser 567. The theoretical findings were validated by *in vitro* enzyme assays. Mol-1 showed potent inhibition of 5-LOX with an IC_50_ value of 4 µM. The overall trend for the binding energies calculated and LUDI scores was in good qualitative agreement with the experimental data. The inhibitory activity of the inhibitor, Mol-1 was further analyzed and confirmed by analysis of products on SP-HPLC. Mol-1 also showed potent anti proliferative effects on MCF-7 cell line (breast cancer) with GI_50 _of 9 μM.


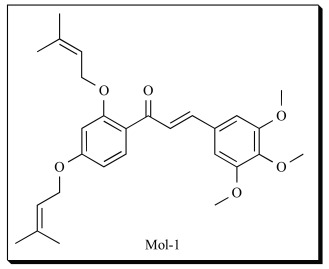


In a recent study, we utilized the crystal structure of human 5-LOX [85]. The availability of reliable structure of 5-LOX encouraged us to perform Structure Based Lead Optimization study of analogs of benzyl propargyl ether, a known class of 5-LOX inhibitors. Mol-2, having IC_50_ of 760 µM [95] was used as the initial molecule for the design studies [44]. The interactions of Mol-2 with 5-LOX active site residues were thoroughly studied to understand the 5-LOX structure better. GOLD docking program was used to dock the inhibitors. After docking, the 5-LOX-Mol-2 complex was analyzed and important amino acids at the active site were identified. Lead optimization studies were manually performed employing Site point connection method. In this study, only the site points corresponding to hydrophobic interactions were considered. As discussed earlier, Du *et al*. (2006) identified that hydrophobic tail plays the very pivotal role in 5-LOX inhibitors along with polar groups. Hence, four site points corresponding to Leu368, Ile 415, Phe421 and Thr364 with potential of forming strong hydrophobic interactions were identified. Modifications were proposed such that all the hydrophobic amino acids can be exploited. The designed molecules were synthesized and further tested *in vitro *for their inhibitory properties against 5-LOX enzyme using the assay described by Reddanna *et al.* (1990) [96]. The number of hydrogen bonds and hydrophobic interactions of the molecules with 5-LOX showed inverse correlation with the experimental IC_50_ values. The most active molecule, Mol-3 inhibited 5-LOX with an IC_50_ value of 8 µM. The molecules also showed good anti-proliferative effects on three different human cancer cell lines; COLO-205 (colonic), HepG_2_ (hepatoma) and MDA-MB-231 (breast). Mol-3 also showed good anti-inflammatory effects *in vivo*. Studies demonstrated the protective effect of Mol-3 in mouse Acute Lung Injury (ALI) model induced by lipopolysaccharide (LPS). 


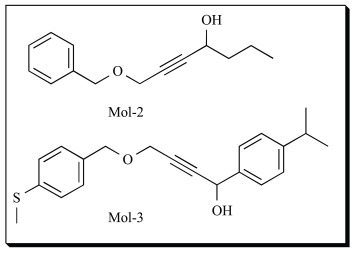


This study is a successful example reported for of 5-LOX inhibitor design using the *de novo* design strategy. There are various other reports employing docking studies to understand structure activity relationship of a class of 5-LOX inhibitors [97-100]. 

## APPLICATION OF LBDD IN 5-LOX INHIBITOR DEVELOPMENT 

### Quantitative Structure Activity Relationship Studies

In the absence of reliable structural information, quantitative structure activity relationship (QSAR) is an alternative approach and one of the widely used ligand based drug design approaches. QSAR is the name given to methods which correlate molecular structure with properties like *in vitro* or *in vivo* biological activity. When applied to toxicological data these methods are termed quantitative structure toxicity relationship (QSTR) and termed quantitative structure property relationship (QSPR) when modeling physicochemical properties [101]. QSAR is based on the assumption that the structure of a molecule (i.e. its geometric, steric and electronic properties) must contain the features responsible for its physical, chemical, and biological activities [102]. QSAR is defined as a process that quantitatively correlates structural molecular properties (descriptors) with functions (i.e. physicochemical properties, biological activities, toxicity, etc.) for a set of similar compounds [103]. The recent advances of QSAR and their applications in drug discovery process have been nicely described by Eleni *et al.* (2003) [102] and Dudek *et al*. (2006) [104]. Flowchart for QSAR methodology has been depicted in Fig. (**3**).

#### QSAR Case Studies

In our recent studies, we performed 3D-QSAR study using CoMFA (Comparative Molecular Field Analysis) and CoMSIA (Comparative Molecular Similarity Index Analysis) techniques on 2-substituted 5-hydroxyindole-3-carboxylate derivatives to determine the influence of steric, electrostatic and hydrophobic fields on their 5-LOX inhibitory activity [105]. Forty compounds with IC_50 _values ranging from 0.031 to 13.4 µM for 5-LOX were selected from the literature [106]. The IC_50_ values were converted into corresponding pIC_50 _values by the formula in Eq. (**2**). The calculated pIC_50_ values ranged from 4.87 to 7.50.

(2) pIC_50_ = - log IC_50_

The initial set of compounds was randomly divided into training set (30 compounds) and test set (10 compounds). All molecular modeling calculations were performed using SYBYL program package version 8.0 (Tripos Associates Inc.) on a Linux operating system [107]. Two datasets of the compounds with different partial charges were prepared; first dataset with charges calculated by the Gasteiger-Hückel method and the second dataset with more advanced methods, the ESPFIT (Electrostatic potential) charges. GAUSSIAN 03 package was used for the generation of second dataset. All the molecules were optimized using HF/6-31G* basis set and the partial atomic charges for each of them were obtained with ESP fitting (HF/6-31G* OPT ESP). CoMFA and CoMSIA models were generated for both the datasets and the results were analyzed. The 3D QSAR models of CoMFA and CoMSIA descriptors were derived using PLS regression method as implemented in the SYBYL package. The cross-validated coefficient, q^2^, was calculated using Eq.** 3**.

(3) $$q^{2}=1-\frac{\sum {\left(Y_{\mathrm{predicted}}-Y_{\mathrm{observed}}\right)}^{2}}{\sum \left(Y_{\mathrm{observed}}-Y_{\mathrm{mean}}\right)}$$

Where Y_predicted_, Y_observed_, and Y_mean_ are predicted, actual, and mean values of the target property (pIC_50_), respectively. $\sum {\left(Y_{\mathrm{predicted}}-Y_{\mathrm{observed}}\right)}^{2}$is the predictive sum of squares (PRESS). 

The LOO cross-validated correlation coefficient q^2^ was obtained with an optimal number of components (N) of six. Among the two datasets with different partial atomic charges, the dataset with ESPFIT charges yielded higher noncross validated r^2^ values (r^2^_ncv_) and cross-validated q^2 ^values (q^2^_cv_). The results clearly showed that the partial atomic charges derived by ESPFIT charges produced better CoMFA and CoMSIA models than the Gasteiger-Huckel charges. The models derived from ESPFIT atomic charges model were further analyzed. 

In the CoMFA electrostatic contour of Mol-4, the hydrogen bond acceptor favored region is present corresponding to the amino acid Gln 557. This corresponded well to the docking interactions. The steric contours showed that steric bulk is favored at the 3´ and 4´ of the phenyl group. The reduced activity of Mol-5 was attributed to the phenyl group on the indole ring. It has been observed that bulky amino acid Phe 421 is present very close, which may be the cause why bulky groups are not favored in that region. The steric contours complemented very well with the docking results. In the CoMSIA contours, hydrogen bond donor unfavorable regions were seen. This correlated well with the pharmacophore model of 5-LOX reported by Aparoy *et al*. recently [108]. The statistical significance of the generated 3D-QSAR models were confirmed using an external set of 10 test set molecules. The predictive correlation coefficient r^2^_pred _was based only on molecules not included in the training set and is computed using the following Eq. **4**


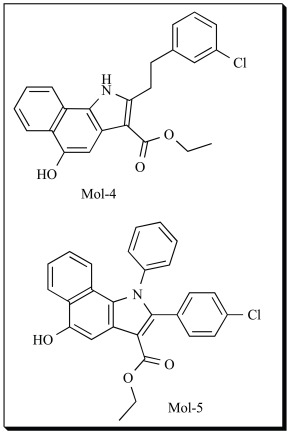

(4) $$r_{\mathrm{pred}}^{2}=\frac{\mathrm{SD}-\mathrm{PRESS}}{\mathrm{SD}}$$

Where SD is the sum of the squared deviations between the biological activity of molecules in the test set and the mean biological activity of the training set molecules and PRESS is the sum of the squared deviations between predicted and actual activity values for every molecule in the test set. 

The predicted activity values of the test set compounds showed good correlation with experimental values, with r^2^_pred_ of 0.661 and 0.713 for CoMFA and CoMSIA models respectively. It indicated that the CoMFA and CoMSIA models of both the datasets could be reliably used to design novel and more potent 5-LOX inhibitors. Hence, the QSAR models were further used to design novel 5-hydroxyindole-3-carboxylate derivatives. Compounds, Mol-6 and Mol-7 are two of the 15 proposed derivatives which may possess higher inhibitory activity towards 5-LOX. In another study, Zheng *et al.* [109] also developed 3D-QSAR model for the same 2-substituted 5-hydroxyindole-3-carboxylates using CoMFA and CoMSIA. They also suggested that hydrogen-bond donor fields can be negligible around the 5-OH of indole ring. They proposed that introduction of bulky groups like isopropyl and t-butyl at 7, 8 positions and electron withdrawing groups like nitro and t-butylamine at the 6-position may further enhance the activity of Mol-4. They also designed few substituents with electron-donating groups such as hydroxymethyl, methoxyl, aminomethyl or methylamine at 5^th^ position. They designed 20 compounds which showed good predicted activity towards 5-LOX inhibition. 


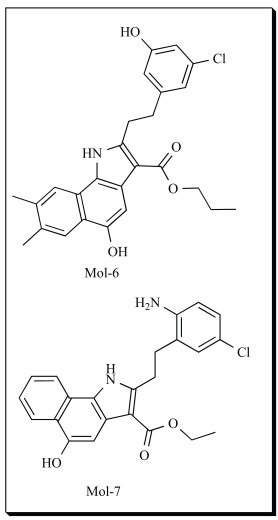


Choudhary *et al.* [110] studied QSAR approach of 4-oxotiazolidines and 5-arylidine derivatives to explore the important physicochemical properties for 5-LOX inhibition. A training set of 22 compounds reported by Yadav R *et al.* [111] were used in the study. From the models developed, they have studied four models which they quoted as best models based on their statistical values. They observed that the steric descriptors, molar refractivity (MR) and connolly accessible area (CAA) and the topological parameters, Balaban index (BI), cluster count (CC), molecular topological index (MTI) and total valence connectivity (TVC) play an important role in 5-LOX inhibition. They concluded that incorporation of bulky groups on thiazolidine nucleus will decrease the binding affinity of 4-oxothiazolidines and increase in branching and presence of hetero atom favors the 5-LOX inhibitory activity. 

Vijay Agrawal *et al. *[112] studied the QSARs for set of 60 1-phenyl [2H]-tetrahydro-triazine-3-one analogues reported by Kim KH *et al*. [113] by using distance-based topological indices. Based on the generated models they suggested the modifications to be made and important features necessary on parent molecule in order to inhibit 5-LOX. It was observed that the lipophilic electron withdrawing substituents at the meta position increased the activity. They concluded that with the increase in size of R_2 _substituent they observed a decrease in the inhibitory activity, the absence of hydrogen at R_5_ and R_5_′, presence of C=O group at R_4_, size, shape and branching are favorable for the 5-LOX inhibition.

In another study, Revathi *et al. *[114] employed QSAR approach on 1, 5-diarylpyrazole analogs, a class of COX-2/5-LOX dual inhibitors. They have taken a set of 10 compounds reported by Pommery *et al. *[115] and ZD-2138 [116] for their studies. From their studies, they demonstrated that the physicochemical properties Hy (hydrophilic factor) and Mor17v (3D molecular representation of structure based on electron diffraction code) are important for more dual COX-2/5-LOX inhibitory activity. Further Mukesh Doble and his group developed QSAR model of seventeen propenone derivatives [117], which were reported to be COX-2/5-LOX dual inhibitors [98]. A training set of 13 compounds was used in their study. The regression model of 5-LOX showed correlation with SC-1, ADME_Absorbtion_T2_ 2D and S_dO. SC-1, which showed positive correlation with 5-LOX inhibition, specifies the number of bonds in the molecule. ADME_Absorbtion_T2_2D also showed positive correlation and indicates the hydrophobicity of the molecule. On the other hand, S_dO specifies number of oxygen atoms with one double bond and it showed negative correlation with 5-LOX inhibition. The developed models showed reasonable predictive capability. 

Arockia Babu *et al*. developed 3D-QSAR model [118] of a series 51 derivatives of chalcones reported by Sogawa *et al*. [119] to identify essential structural and physicochemical sites required for binding to 5-LOX. APEX-3D system was used for their study. In their study, two models with three biophoric sites and four secondary sites showed good correlation. They concluded their study proposing various substitutions that can further increase 5-LOX inhibitory activity.

In an earlier study in 1990, Summers *et al*. [120] performed QSAR studies for 111 hydroxamic acids and identified that hydrophobicity of the molecule influences the *in vitro* 5-LOX inhibitory potencies of the compounds. In their study, they developed four models by dividing the compounds into four structural groups, namely arylhydroxamic acids, arylacrylohydroxamic acids, (arylalkyl) hydroxamic acids and [(aryloxy)alkyl] hydroxamic acids. They observed a good correlation between the octonal-water partition coefficient of the group attached to the carbonyl of the hydroxamate and the potency for 5-LOX inhibition. Importantly, they revealed that hydrophobic groups close to the hydroxamic acid functionality do not contribute to increased inhibition and the hydrophobicity of fragments beyond approximately 12 Å from the hydroxamate do not influence potency. They also demonstrated that inhibitory activity was enhanced in cases where there was an alkyl group on the hydroxamate nitrogen, electron-withdrawing substituents and when the hydroxamate was conjugated to an aromatic system. These results provided a simple and a very reasonable description of the hydroxamic acid binding site in 5-LOX.

### Pharmacophore Modeling Strategies

Paul Ehrlich introduced the pharmacophore concept in the early 1900s and Ehrlich suggested the term *pharmacophore *to refer to the molecular framework that carries (*phoros*) the features that are essential for the biological activity of a drug (*pharmacon*) [121]. Later in 1977, Peter Gund defined it as “a set of structural features in a molecule that are recognized at the receptor site and is responsible for that molecule’s biological activity” [122]. Recently, pharmacophore based virtual screening has become a very useful tool for hit identification stage of drug development. It has become the major tool in drug design studies where the three-dimensional (3D) structure of the target is unknown. The main advantage of this approach is rapid screening of millions of compounds for identification of potential candidates. Pharmacophore mapping/modeling involves three processes: (i) finding the features required for a particular biological activity (ii) determining the molecular conformation required (i.e. the bioactive conformation); and (iii) developing a superposition or alignment rule for the series of compounds. The process of pharmacophore based virtual screening encompasses sequential computational steps: drug target selection, database preparation, pharmacophore model generation, 3D screening [123-127]. Automated pharmacophore mapping is now available in programs like Catalyst [128], LigandScout [129], DISCO [130], GASP [131], Phase [132], MOE [133], etc. The typical types of interaction sites recognized by pharmacophore software include hydrogen bond acceptor (A), hydrogen bond donor (D), hydrophobic (H), negative ionic/ionizable (N), aromatic rings (R) and positive ionic/ionizable (P). All these methods use activity for development of pharmacophore models that can distinguish actives and inactives, while discarding those that do not.

#### Pharmacophore Modeling Case Studies

There are various reports where pharmacophore modeling was employed to understand the important features for 5-LOX inhibitors. Charlier* et al*. (2006) have combined ligand based and target based approaches to elucidate the structural insights of human 5-LOX [87]. In their study, a pharmacophore model was generated and was explained together with homology model of 5-LOX. The pharmacophore model was generated using the non redox class of inhibitors. These active-site directed inhibitors have been shown to be more potent and selective towards 5-LOX [134]. Till to date, there are few limited reports on non-redox inhibitors of 5-LOX. As the range of 5-LOX inhibition activities of the inhibitors of these classes was limited the authors did not generate quantitative structure-activity relationships. The selected molecules represent a pool of potent inhibitors acting on the same target with a similar mechanism (non redox). Therefore, they applied the HipHop process from Catalyst software, which compares diverse and highly active inhibitors to derive 3D hypotheses based on common chemical features identified on the superposition of active compounds, without considering biological activities. 

The pharmacophore model of 5-LOX developed using HipHop process from Catalyst software, is comprised of two hydrogen bond acceptors, two hydrophobic groups and an aromatic ring. The model was based on non-redox inhibitors of the following chemical families (i) the thiopyranindole derivatives [135], (ii) the biaryls [136] and finally (iii) imidazole [137] compounds. Structures of five of the training set molecules are shown as Mol-8 to Mol-12. Further, in their study, they developed the 3D structure of 5-LOX by homology modeling based on the crystal structure of rabbit 15-LOX [138]. In agreement with mutagenesis studies, the modeled active site of human 5-LOX was found to be more spacious than that of rabbit 15-LOX [139]. The 5-LOX active site was then characterized from a structural point of view and used to study the docking of selected representative inhibitors. The pharmacophore was fitted into the active site of 5-LOX and the important interactions were explained. The important anchoring points identified inside the binding cavity include Tyr181, Leu414, Asn425, Arg411 and Phe421 that could form favorable interactions with ligands. The study was a first step towards the characterization of human 5-LOX and its interaction with ligands.

As discussed earlier Catalyst/HipHop generates hypotheses based on only the identification and overlay of common features using known active ligands, and not activity data whereas the Catalyst/HypoGen pharmacophore model identifies chemical functional features that are typical of active compounds, thus facilitating their differentiation from inactive compounds [128]. Hence, HypoGen is regarded as a quantitative pharmacophore modeling approach and HipHop is regarded as a qualitative approach. The HypoGen modeling requires a training set with structural diverse inhibitors covering a wide range of activities of at least four orders of magnitude whereas in the case of the HipHop pharmacophore modeling, a small ligand set with highly active compounds is sufficient to get a reasonable model. However, a HipHop model cannot be further applied to predict the activity value of the virtual hit; generally a large number of false positive hits are observed in the case of HipHop pharmacophore-based virtual screening [140].

In an attempt to develop a reasonable pharmacophore model which can be successfully applied to virtual screening we generated a 5-LOX pharmacophore model using Catalyst/HypoGen approach along with Catalyst/HipHop (Aparoy *et al*, 2010) [110]. All the pharmacophore modeling calculations were carried out by using the Catalyst 4.11 software package (Accelrys, San Diego, USA) on SGI workstation. 89 reported 5-LOX inhibitors from 18 different studies were considered in the study. The pharmacophore model was developed using HypoGen module from a training set of 24 compounds with IC_50_s ranging from 0.003 µM to 41 μM for 5-LOX. Before performing the quantitative pharmacophore modeling, the qualitative HipHop model was generated based on the five most active compounds in training set, the purpose of which was to identify pharmacophore features necessary for potent 5-LOX inhibitors. The HipHop pharmacophore hypothesis indicated the importance of hydrogen-bond acceptor, hydrogen-bond acceptor lipid, hydrogen-bond donor, hydrophobic moiety, hydrophobic aliphatic moiety, hydrophobic aromatic moiety and ring aromatic feature.


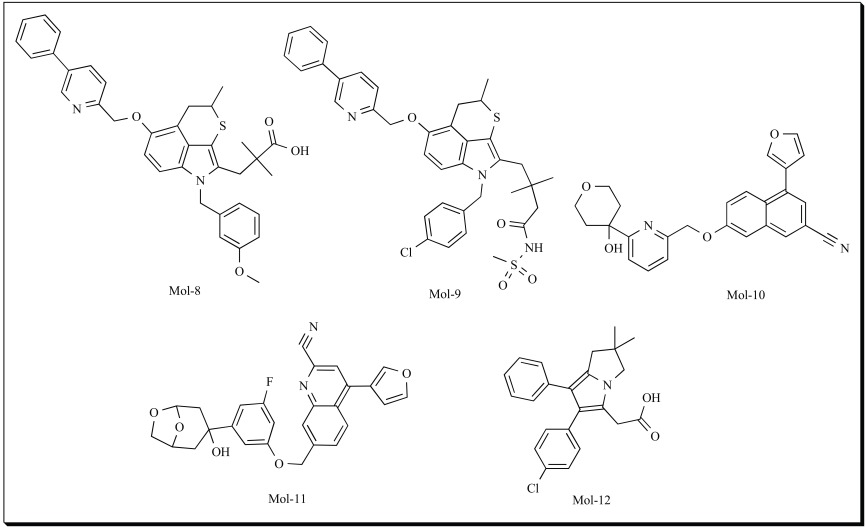


Furthermore, we developed a quantitative pharmacophore model using the HypoGen module of Catalyst which can be used to correlate the observed biological activities for a series of compounds with their chemical structures. The generated HypoGen models were evaluated according to Debnath in terms of cost functions and statistical parameters. Three cost parameters, namely, null cost, fixed cost and total cost are generated during pharmacophore modeling. A pharmacophore model should have a high correlation coefficient, lowest total cost and RMSD (Root Mean Square Deviation) values. The total cost should be close to the fixed cost and away from the null cost. The total cost of each pharmacophore is computed by the sum of three costs: weight, error and configuration. The total cost parameter results in a ranking of generated hypothesis. This parameter takes into account the correlation of the training set molecules, tested activities with the activity estimated by the hypothesis. The fixed cost parameter assumes all training set molecules fit the simplest possible hypothesis perfectly. This cost parameter has the smallest numerical value of all the hypotheses cost parameters. The null cost parameter assumes that all training set molecules have the same activity, so that there is no statistically significant structure in the training set. This cost parameter has the highest numerical value of all the cost parameters. The difference between the cost of the generated hypothesis and the cost of the null hypothesis signifies the reliability of a pharmacophore model. A value of 40–60 bits between them for a pharmacophore hypothesis may indicate that it has 75–90% probability of correlating the data. 

The 5-LOX pharmacophore model developed by Aparoy *et al*., 2010 [110] was characterized by the lowest total cost value (108.338), the highest cost difference (58.281) and contains four features, namely, two hydrogen-bond acceptor, one hydrophobic and one ring aromatic feature. A ‘measured’ versus ‘estimated’ activity for the training set exhibited a correlation coefficient (*r*) of 0.974978 with RMSD of 0.6025. The good score value indicated a reliable ability to predict activities within the training set. The model was validated by Fischer randomization test method by using the CatScramble program implemented in Catalyst [141]. 

The ability of the pharmacophore model generated to predict activities of 5-LOX inhibitors was evaluated using an independent test set which contains 65 external compounds. The experimental and predicted activities of the test set compounds were compared and a fairly good correlation coefficient of 0.85 was observed for regression analysis of the experimental and predicted inhibitory activity values. The validation results proved the accuracy of the model generated and hence it was further used for virtual screening process. The best quantitative pharmacophore model generated was used as a 3D query to screen several commercial databases comprising of compounds. The virtual screening procedure applies has been well depicted in Fig. (**4**). Three chemical databases (Enamine, NCI, Maybridge) containing two million compounds in total were screened utilizing the 5-LOX pharmacophore model developed as a query and 15,162 unique structures (8% of all virtually screened compounds) were able to match the pharmacophore model. The top 5000 molecules were further docked into 5-LOX active site using GOLD software. Most of the molecules showed positive Gold Score and were ranked using GoldScore function. The top 1000 molecules were complexed with 5-LOX and the protein-ligand interactions were scored using LUDI and Ligandfit in Accelrys Discover Studio [142]. Since there is no generally accurate and reliable scoring function so far, the compounds which were commonly scored top by various applications were ranked higher. After screening by visualization of protein–ligand interactions, hundreds of potential compounds were identified. Five compounds which were readily available were procured and tested for their 5-LOX inhibitory activity. Two compounds, Mol-13 and Mol-14 showed inhibition with IC_50 _values 14 µM and 35 µM respectively. The biological evaluation further supported the model generated and illustrated its importance in identification/development of novel 5-LOX inhibitors. It should be noted here that the tested compounds were not the best among the hits identified in the screening. As the compounds were readily available they were given preference in the study. The anti-cancer/anti-proliferative effects of the Mol-13 and Mol-14 were further estimated in cancer COLO-205 (colon cancer) cell line. Both compounds showed anti-proliferative effects in a dose dependent manner, with GI_50_ of 46.5 µM and 67.0 µM respectively [91].

In another study, Sukesh Kalva *et al. *[143] also employed pharmacophore/docking based virtual screening approach and proposed 6 compounds which may inhibit 5-LOX. They have taken 102 structurally diverse compounds from literature and divided them into a training set of 22 compounds and a test set of 80 compounds. Training set of 22 compounds was used to develop the pharmacophore model using HypoGen module present in Catalyst. The best model has two hydrogen bond acceptors and two hydrophobic groups. They have used their model to screen the database and retrieved 3000 compounds which they further narrow downed to 220 based on cluster analysis. The inhibitors were docked into the modeled 5-LOX to understand the binding interactions of proposed compounds and to correlate these interactions with pharmacophore features. The identified compounds can be used further for biological validation studies.

In all the pharmacophore models of 5-LOX reported it has been identified that two hydrogen bond acceptors, hydrophobic and aromatic are common, hence, it can be concluded that these interactions play important role in 5-LOX inhibition. The pharmacophore methods proposed showed good promise and can be further applied in lead identification process.

### Scaffold Hopping Case Studies

Scaffold hopping is a technique which aims to find compounds that are structurally diverse and share a particular biological activity [144] that is, preserving the 3D interaction properties of a scaffold while changing the structural skeleton [145]. If structurally diverse compounds are identified, this would help in finding new classes of compounds against the target protein [146]. It has been described by Renner and Schneider as “finding isofunctional but structurally dissimilar molecular entities” [147]. There are few automated methods available for perform scaffold hopping. Gisbert Schneider and his group have applied this technique effectively in the development of novel inhibitors of 5-LOX. Some of their findings are reviewed in this article.


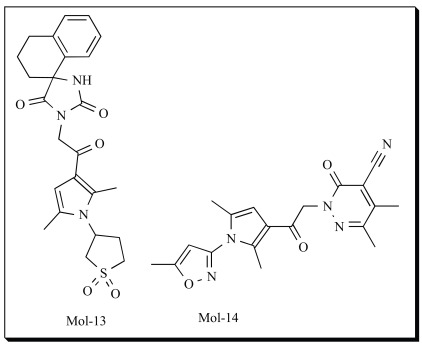


Hofmann *et al*. (2008) have applied Scaffold-Hopping to yield potent inhibitors of 5-LOX. Ligand-based virtual screening methods were used in an iterative fashion to identify new inhibitors [148]. The study consisted of four subsequent cycles of virtual screening, including 3D- and 2D-based methods and substructure searching. Eleven dual 5-LOX/COX reference inhibitors were selected from the literature. In this study, low energy conformers of the 11 molecules were generated using CORINA and partial atomic charges were computed with PETRA (both from Molecular Networks GmbH, Erlangen, Germany). The low energy conformer of each molecule was further used for descriptor calculation. Scaffold hopping technique was applied to retrieve isofunctional chemotypes with different backbone architecture from a large compound collection. Two similarity search methods, “Charge3D” and “TripleCharge3D” were used to perform alignment-free similarity searches against Asinex Gold (November 2005: 231,812 compounds) and Platinum (132,250 compounds) collections (Asinex Ltd., Moscow, Russia) for each of the 11 queries. Two virtual screens applying Charge3D and TripleCharge3D were performed using each of the 11 reference molecules. The ten top-ranked compounds of each of the 22 virtual screens were shortlisted and compounds detected by both methods were further tested for their *in vitro* activity. Out of the top virtual screen hits 6 molecules showed good activity of IC_50 _less than 15 µM. Further, in a second selection round, one of the virtual screening hit, compound Mol-15 (IC_50_ of 6 µM) was used for a similarity search using MACCS substructure keys of Molecular Operating Environment (MOE) molecular modelling software package [133]. The MACCS structural fingerprints have 166 bits each indicating the presence of a predefined substructure or functional group. The degree of similarity of two structures is thereafter established by calculating the Tanimoto coefficient. Again, the Asinex Gold compound collection was screened, and compounds with a Tanimoto coefficient > 0.85 (indicating high structural similarity to compound, Mol-15) were identified. From the potential hits retrieved twelve commercially available compounds were manually selected and tested in whole cell assays. Among the pyridine-imidazoles tested, compounds Mol-16 and Mol-17 were most potent, with IC_50 _of 1.3 and 0.9 µM respectively. In third selection round, the influence of various substitutions on pyridine-imidazole ring was studied retaining the phenyl-dimethylamino-motif. Again, the Asinex Gold compound collection was screened using compounds Mol-16 and Mol-17 as query. Five potential hits were obtained out of which one molecule, compound Mol-18, had IC_50_ of 0.6 µM. The position of the methyl group on the imidazole ring did not significantly affect ligand potency. Finally, in fourth round of virtual screening the scaffold of the pyridine–imidazole structure was replaced. The shape- and“fuzzy” pharmacophore-based technique SQUIRREL [149] was applied to find candidates in the Asinex Platinum compound collection. From the potential candidates identified 5 compounds were evaluated *in vitro*. In the study, Schneider and co-workers demonstrated that single round of pharmacophore-based compound ranking is insufficient to identify potent hits and a multistep virtual screening procedure methods including structure based similarity searching is more accurate and productive in retrieving new scaffolds and potential 5-LOX inhibitors. One of the hits obtained from the study, Mol-19 was further employed for scaffold hopping and a novel potential inhibitor of 5-LOX, Mol-20 (IC_50_ of 0.09 µM) was developed and the study has been recently published [150]. 


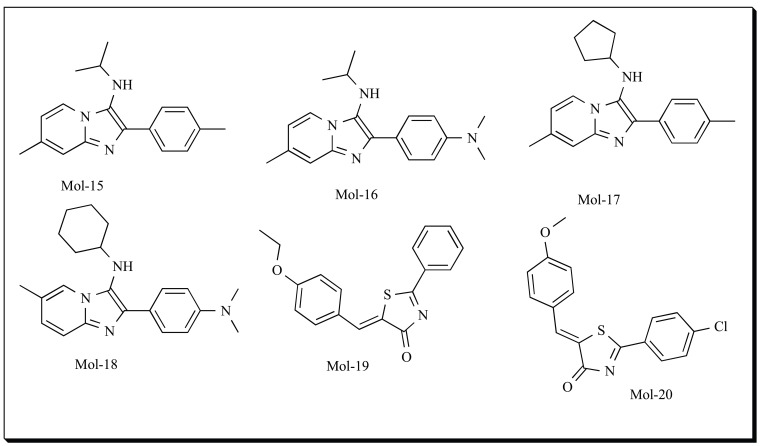


In one of their earlier reports in 2007, Schneider and coworkers identified natural product derived inhibitors of 5-LOX by ligand based virtual screening. A diverse set of known 5-LOX inhibitors was used as query in a database scan for alternative chemotypes with 5-LOX inhibitory activity [151]. This ligand-based virtual screening approach consisted of two consecutive steps: First, 43 queries taken from a collection of published bioactive compounds (COBRA collection, version 4.1) [152] were used for screening natural products and natural product derived combinatorial compound collections from the AnalytiCon Discovery compound repository. Software speedCATS was used in the study [153-154]. From the promising hits obtained, 18 molecules were randomly selected and tested for their inhibitory activity towards 5-LOX. The two most active molecules from round 1 (with IC_50_ of 1-10 µM) were further used as queries for similarity searching in second round of virtual screening. 4 different virtual screening methods were used (three variants of the CATS approach [153] and MACCS keys with the Tanimoto index [155]). 17 compounds were selected and screened. The study resulted in the identification of two novel chemotypes with nanomolar range activity in intact polymorphonuclear leukocytes (PMNL). Compounds, Mol-21 and Mol-22 are some of the inhibitors identified. 


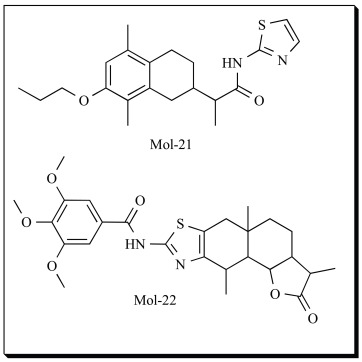


### Pseudoreceptor Modeling Case Studies

Pseudoreceptor modeling is a new concept in computer aided drug design which allows the reconstruction of the three-dimensional structure of an unknown target based on the structures of its ligands (known bioactive compounds). It combines the present techniques but significantly extends their possibilities by the generation of an explicit receptor model. This model may subsequently be used for affinity prediction and other receptor-based modeling tasks [156]. Pseudoreceptor models bridge the gap of ligand- and receptor-based drug design. Pseudoreceptor models fall into the class of 3D QSAR methods in computational chemistry. A recent comprehensive review by Schneider *et al*. [157] has covered the theoretical background and described the examples of pseudoreceptor model based drug design studies. 

Pseudoreceptor modeling has been successfully applied by Hofmann and his colleagues for the identification of novel inhibitors of 5-LOX [158]. A series of imidazo-[1,2a]-pyridines identified as highly potent 5-LOX inhibitors from Scaffold Hopping studies were used for the development of pseudoreceptor model of 5-LOX binding site. “Flexible alignment” routine of MOE 2007.09 software was used. Pseudoatoms that delineate putative receptor atom positions were projected around ligand interaction sites (potential pharmacophoric points). Three such pharmacophoric features (hydrogen-bond acceptor, hydrogen-bond donor, п stacking [aromatic]) were considered. Compounds from the SPECS database (202 681 compounds), Asinex gold (229 398 compounds) and platinum (125 231 compounds) were used for screening. Single low energy conformer of the compounds was generated using the CORINA 3.2 software. Then the structures were prepared for pseudoreceptor descriptor calculation using the so-called “washing” routine (deletion of salts and de/protonation of strong acids and bases) of the (MOE) software. The compounds were screened using the pseudoreceptor model generated. Compounds with a common physicochemical profile were identified and 14 compounds with different heteroaromatic scaffolds were manually selected. These compounds were ordered and subsequently tested for 5-LOX inhibition. Of the 14 compounds tested, 11 were able to inhibit 5-LOX at 10 µM, and of these 11, eight did so with an IC_50_ value equal or less than 10 µM. The most potent compound (Mol-23), a fused heterocycle derived from benzo[a]phenazine showed an IC_50 _of 0.96 µM. These results encourage the use of pseudoreceptor modeling for virtual screening studies.


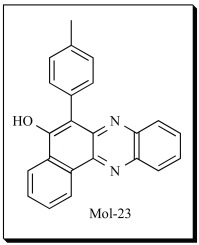


### Limitations of the Current Drug Design Approaches Towards 5-LOX

As pointed out by Rödl *et al*. [158] the crystal structure of 5-LOX reported recently represents an apoprotein structure without a bound ligand. Hence, this structure may have its limitations in structure based inhibitor design approaches. Advanced, structure refinement like molecular dynamic (MD) simulations should be employed to 5-LOX-inhibitor complexes to get reliable results. For the time being, hybrid approaches like pseudoreceptor modeling are being employed to bridge structure based and ligand based approaches, but they have their limitations. The lack of experimental evidence of binding modes of different types of LOX inhibitors i.e. redox, non-redox and non-specific make CADD approaches more challenging. 

The accuracy of the current CADD approaches for 5-LOX can be improved enormously by the study of protein flexibility, induced-fit adaptations, the role of water insolvation, desolvation and ligand binding. One of the major limitations in drug design process is the inability of the existing docking programs to accurately estimate binding affinities. Combination of docking protocol and scoring function to be employed requires validation before their application in a drug discovery project. In virtual screening processes, it is noticeable that some of the high scoring ligands miss interactions that are known to be important for the target receptor [159]. Improving the predictive accuracy of scoring functions in various docking programs is a major challenge to computational chemists and it would help these methods to enable a greater impact in lead identification and optimization stage of drug discovery. Better understanding of details of the electrostatics at 5-LOX binding site and changes in protonation states at the iron binding site will help in better planning of SBDD experiments [160]. Quantum chemistry methods could be helpful in understanding these molecular details. 

The most important challenge for pharmacophore based virtual screening is the percentage of virtual hits that are really bioactive. Usually the screening results bear a higher false positive and false negative rate. Many factors can contribute to this problem, including the quality and composition of the pharmacophore model and the available information of 5-LOX structure. The increase in number of potential inhibitors of 5-LOX, with activities derived from a uniform assay method would surely enhance the quality of these methods.

From the case studies discussed in the review it is evident that the main focus has been on the development or identification of potential 5-LOX inhibitors. There has been limited attention on the development of isozyme specific 5-LOX inhibitors. As discussed, most of the potent inhibitors of 5-LOX identified from *in vitro* studies showed toxic side effects in animal studies or human clinical trials. This may be due to non-specific binding to other LOX isoforms, 12-LOX and 15-LOX. LOXs have a conserved structural pattern, mainly at the catalytic site hence non-specific binding will remain a major issue. CADD approaches should be applied to understand the molecular details of the binding sites of LOX isoforms and salient features/patterns should be illustrated to further exploit in specific inhibitor design. With the availability of crystal structures of 5-LOX [85], 12-LOX and 15-LOX [138], methods like receptor based pharmacophore can provide more structural insights into isoform specific LOX activity. The main application of CADD is not only direct identification/development of drugs but also providing information like these in parts which would provide rationality to the drug development process, especially in fields like structure based virtual screening. With more information on binding site features important for selectivity of these LOX isoforms, inhibitors that are specific and effective can be developed and these may cause fewer or no side effects.

## CONCLUSION

5-LOX is the key enzyme involved in the biosynthesis of leukotrienes, the mediators of allergy, asthma, GERD, Crohn's disease and other inflammatory disorders. 5-LOX is also associated with various cancers. As 5-LOX is implicated in many diseases, there is growing emphasis by many pharmaceutical companies and academic research groups on the development of effective 5-LOX inhibitors. Structure based and ligand based drug design approaches are being employed in 5-LOX drug development strategies. Lack of crystal structure information of 5-LOX, however, has been an obstacle for the application of structure based drug design strategies. Homology models of lipoxygenase enzymes have been used in several previous studies but there are fewer reports on their usage for inhibitor discovery. Specifically, homology models were sometimes used effectively to understand SAR of novel class of inhibitors by docking methods. There are few reports where homology model of 5-LOX has been used further in designing novel inhibitors. These studies resulted in the design of novel inhibitors with μM range activity. The recent elucidation of the crystal structure of 5-LOX should advance the 5-LOX inhibitor design in the coming years. It can be a working tool for more precise predictions of function and binding affinities of inhibitors. 

Ligand based drug design strategies have been widely employed to quantitatively explore common chemical characteristics among a considerable number of known 5-LOX inhibitors with great diversity. Results of pharmacophore modeling studies clearly suggest the importance of hydrogen bond acceptor, hydrophobic and aromatic interactions for 5-LOX inhibitory activity. Successful application of these pharmacophore models as a query for searching chemical databases and identification of new chemical entities supports their possible use in the assessment of binding affinities. The application of pseudoreceptor models and scaffold hopping in computer aided 5-LOX inhibitor design is also well demonstrated. Scaffold hopping will aid in finding new classes of compounds against 5-LOX and should be a main focus. CADD approaches have been so far applied in the development of specific 5-LOX inhibitors, however, in future more emphasis should be on the design of isozyme specific inhibitors which may have fewer or negligible side effects.

## Figures and Tables

**Fig. (1):**
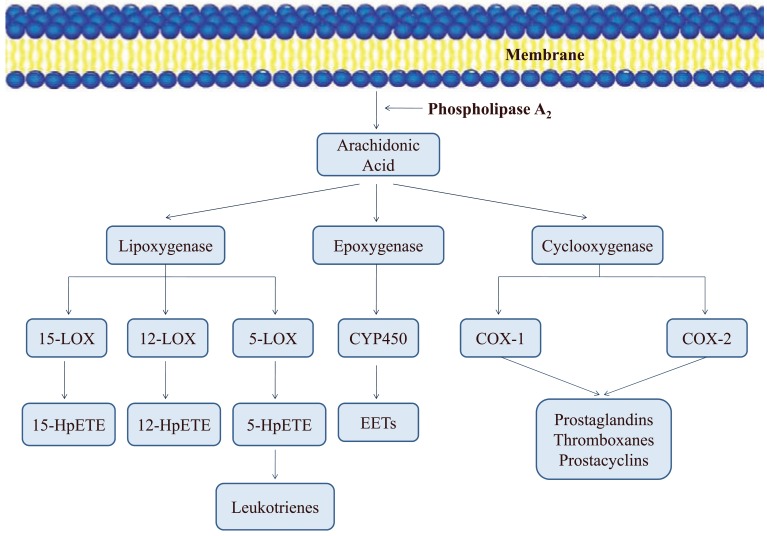
**Overview of Arachidonic acid metabolism in mammalian systems.** AA can be metabolized *via* three major pathways, namely the Lipoxygenase pathway, Epoxygenase pathway and the Cyclooxygenase pathway.

**Fig. (2):**
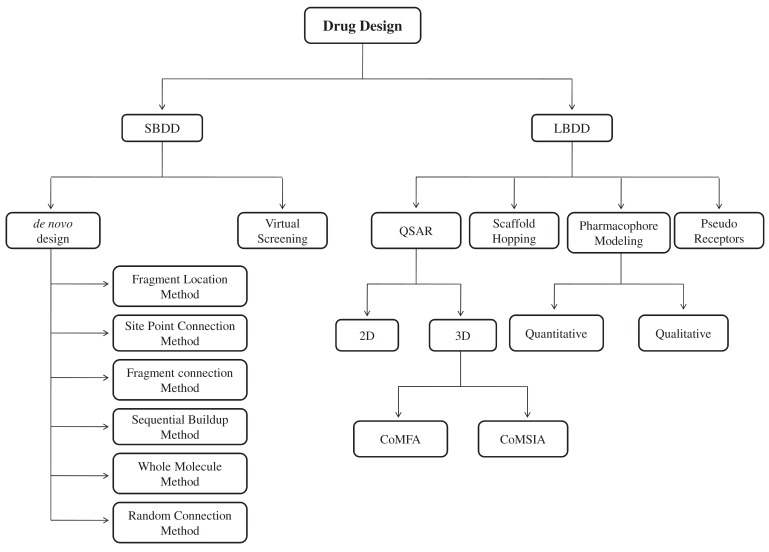
**Basic principles and types of drug design.** The term SBDD explicates various approaches wherein the structural knowledge of the drug target is exploited and LBDD explains the strategies wherein the information from existing ligands of a drug target is utilized.

**Fig. (3):**
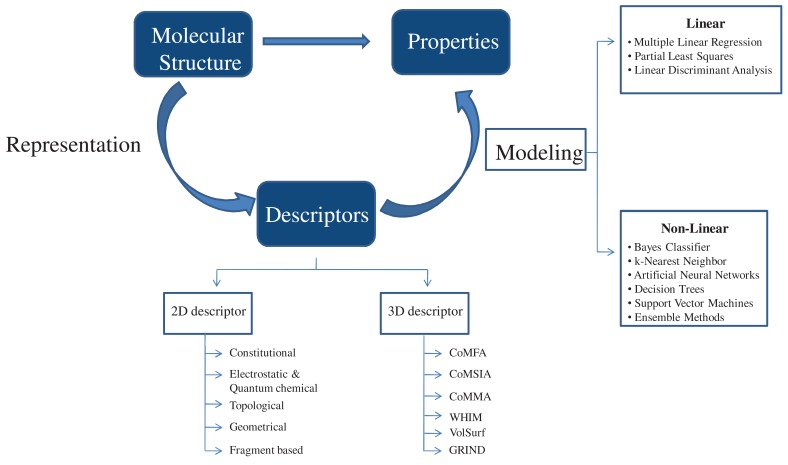
**General methodology of a QSAR study.** QSAR is the process of studying a series of molecules of different structure and properties and attempting to find empirical relationship between structure and property.

**Fig. (4):**
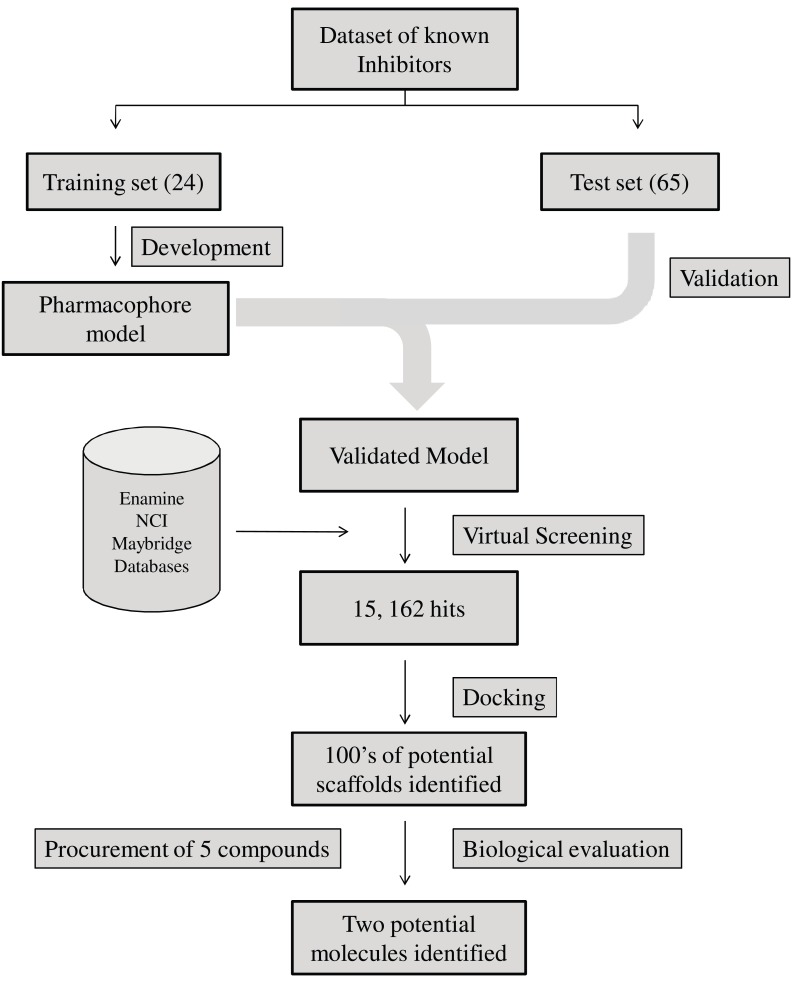
**Virtual screening methodology employed by Aparoy *et al*. [104].** Example of a typical a combined Pharmacophore/Docking based virtual screening approach wherein the advantages of both docking based and pharmacophore based virtual screening can be fully utilized.
